# Germline Mutations in Cancer Predisposition Genes are Frequent in Sporadic Sarcomas

**DOI:** 10.1038/s41598-017-10333-x

**Published:** 2017-09-06

**Authors:** Sock Hoai Chan, Weng Khong Lim, Nur Diana Binte Ishak, Shao-Tzu Li, Wei Lin Goh, Gek San Tan, Kiat Hon Lim, Melissa Teo, Cedric Ng Chuan Young, Simeen Malik, Mann Hong Tan, Jonathan Yi Hui Teh, Francis Kuok Choon Chin, Sittampalam Kesavan, Sathiyamoorthy Selvarajan, Patrick Tan, Bin Tean Teh, Khee Chee Soo, Mohamad Farid, Richard Quek, Joanne Ngeow

**Affiliations:** 1 Cancer Genetics Service, Division of Medical Oncology, National Cancer Centre, Singapore, 169610 Singapore; 2 Centre for Computational Biology, Duke-NUS Medical School, Singapore, 169857 Singapore; 3 Singhealth Duke-NUS Institute of Precision Medicine (PRISM), Singapore, 169856 Singapore; 4 Division of Medical Oncology, National Cancer Centre, Singapore, 169610 Singapore; 5 Department of Molecular Pathology, Singapore General Hospital, Singapore, 169610 Singapore; 6 Department of Anatomical Pathology, Singapore General Hospital, Singapore, 169610 Singapore; 7 Division of Surgical Oncology, National Cancer Centre, Singapore, 169610 Singapore; 8 Laboratory of Cancer Epigenome, Division of Medical Sciences, National Cancer Centre, Singapore, 169610 Singapore; 9 Cancer & Stem Cell Biology Program, Duke-NUS Medical School, Singapore, 169857 Singapore; 10 Department of Orthopaedic Surgery, Singapore General Hospital, Singapore, 169610 Singapore; 11 Division of Radiation Oncology, National Cancer Centre, Singapore, 169610 Singapore; 12 Cancer Science Institute of Singapore, National University Singapore, Singapore, 169610 Singapore; 13 Division of Molecular and Cellular Research, National Cancer Centre, Singapore, 169610 Singapore; 14 Institute of Molecular and Cellular Biology, A*STAR, Singapore, 138673 Singapore; 15 Oncology Academic Clinical Program, Duke-NUS Medical School, Singapore, 169857 Singapore; 16 Lee Kong Chian School of Medicine, Nanyang Technological University, Singapore, 639798 Singapore

## Abstract

Associations of sarcoma with inherited cancer syndromes implicate genetic predisposition in sarcoma development. However, due to the apparently sporadic nature of sarcomas, little attention has been paid to the role genetic susceptibility in sporadic sarcoma. To address this, we performed targeted-genomic sequencing to investigate the prevalence of germline mutations in known cancer-associated genes within an Asian cohort of sporadic sarcoma patients younger than 50 years old. We observed 13.6% (n = 9) amongst 66 patients harbour at least one predicted pathogenic germline mutation in 10 cancer-associated genes including *ATM*, *BRCA2, ERCC4, FANCC, FANCE, FANCI, MSH6, POLE, SDHA* and *TP53*. The most frequently affected genes are involved in the DNA damage repair pathway, with a germline mutation prevalence of 10.6%. Our findings suggests that genetic predisposition plays a larger role than expected in our Asian cohort of sporadic sarcoma, therefore clinicians should be aware of the possibility that young sarcoma patients may be carriers of inherited mutations in cancer genes and should be considered for genetic testing, regardless of family history. The prevalence of germline mutations in DNA damage repair genes imply that therapeutic strategies exploiting the vulnerabilities resulting from impaired DNA repair may be promising areas for translational research.

## Introduction

Sarcoma comprises a family of mesenchymal tumors that arise predominantly in the bone or soft tissue. They are heterogeneous with over 70 histological subtypes^1, 2^, develop from various anatomical sites and can affect patients across age groups^3^. Sarcomas account for 1% of adult solid tumors and nearly 21% of pediatric solid tumors, and carry a poor prognosis, primarily due to late diagnosis and advanced disease at presentation^3^. Although mostly sporadic in occurrence, sarcoma has also been associated with hereditary cancer syndromes^2, 4^. A primary example is Li-Fraumeni Syndrome (LFS), an autosomal-dominant cancer syndrome associated with germline *TP53* mutations. LFS is characterized by a tumor spectrum that includes sarcoma, typically developing before age 45 years^4, 5^. In hereditary retinoblastoma, patients with germline *RB1* mutations are at increased risk of second primary tumors comprising mostly sarcomas^2, 4^. These associations of sarcoma with cancer syndromes implicate genetic predisposition in sarcoma development. However, given the heterogeneity and rarity of sarcomas, few studies have investigated genetic susceptibility in sporadic sarcoma. A greater understanding of genetic predisposition in sarcoma development will help refine our interpretation on the clinical implications of genetic alterations to sarcomas as well as facilitate identification of sarcoma patients who may be at risk for other cancers. This will guide patient-care strategies, such as offering predictive testing for cancer syndromes and preventive surveillance.

Few studies to-date have been published regarding germline alterations in sarcomas^6–11^; those that did mostly focused on specific sarcoma subtypes, such as Ewing’s sarcoma^8^, rhabdoid tumors^10, 11^ and osteosarcoma^9^. The largest study to-date was performed by the International Sarcoma Kindred Study (ISKS), in which a predominantly kindred-oriented cohort of 1192 sarcoma probands were interrogated for germline mutations in a panel of cancer-associated genes. They reported that 55% of sarcoma cases harboured at least one pathogenic mutation^7^. This is a strikingly large proportion, although it should be noted that as a kindred study, their cohort is likely to bias for individuals with familial history for cancer and thus, potential germline mutation carriers. However, it is unknown whether findings from a predominantly Caucasian cohort extrapolate to an Asian population. To address this, we interrogate an Asian cohort of sarcoma patients in this study for the prevalence of germline alterations in 52 cancer-associated genes using a combined approach of targeted genomic sequencing and digital multiplex ligation-dependent probe amplification (digitalMLPA).

## Results

### Patient characteristics

In our sequenced Asian cohort of 66 sarcoma patients aged less than 50 years, 50 were Chinese (75.7%), 4 Indian (6.1%), 1 Malay (1.5%) and 11 (16.7%) were of other ethnicities (Table 1). Male-to-female ratio was approximately balanced at 47%:53%. Age-at-diagnosis ranged between 16–49 years, with a median of 39 years. The cohort included patients of 27 sarcoma histological subtypes (Table 1). Leiomyosarcoma was the most frequent subtype (n = 8, 12.1%), followed by undifferentiated pleomorphic sarcoma (n = 7, 10.6%), Ewing’s/PNET (primitive neuroectodermal tumor) (n = 6, 9.1%) and epitheloid sarcoma (n = 5, 7.6%).

**Table 1:** Characteristics of the sarcoma cohort sequenced in this study.

Characteristics		Cohort size (n = 66) No. patients	%			
Ethnicity	Chinese	50	75.7			
	Indian	4	6.1			
	Malay	1	1.5			
	Others	11	16.7			
Sex	Male	31	47.0			
	Female	35	53.0			
Age at diagnosis	Range	16–49 years				
	Median	39 years				
				**Age at diagnosis years**		**Sex**
Histology				**Range**	**Median**	**Male: Female**
	Leiomyosarcoma	8	12.1	18–49	41	4: 4
	Undifferentiated pleomorphic sarcoma	7	10.6	29–48	37	3: 4
	Ewing’s/PNET	6	9.1	16–43	26.5	4: 2
	Epitheloid sarcoma	5	7.6	21–48	24	2: 3
	Solitary fibrous tumor	5	7.6	38–45	43	1: 4
	Synovial sarcoma	4	6.1	26–49	37.5	2: 2
	Liposarcoma (well / de-differentiated)	4	6.1	34–43	42.5	2: 2
	Liposarcoma (myxoid)	2	3.0	38–43	40.5	2: 0
	Liposarcoma (unspecified)	1	1.5	32	32	0: 1
	Alveolar rhabdomyosarcoma	3	4.5	24–49	43	1: 2
	Spindle cell rhabdomyosarcoma	1	1.5	32	32	1: 0
	Alveolar soft part sarcoma	2	3.0	21–28	24.5	0: 2
	Dermatofibrosarcoma protruberans	1	1.5	37	37	1: 0
	Giant cell tumor of bone	2	3.0	16–47	31.5	1: 1
	Myxofibrosarcoma	2	3.0	40–48	44	2: 0
	Osteosarcoma	2	3.0	20–49	34.5	1: 1
	Chondrosarcoma (clear cell)	1	1.5	33	33	0: 1
	Clear cell sarcoma	1	1.5	43	43	0: 1
	Desmoid/Fibromatosis	1	1.5	22	22	0: 1
	Diffuse type giant cell tumour (pigmented villonodular tenosynovitis)	1	1.5	17	17	1: 0
	Endometrial stromal sarcoma	1	1.5	47	47	0: 1
	Fibrosarcoma	1	1.5	49	49	0: 1
	Inflammatory myofibroblastic tumor	1	1.5	23	23	1: 0
	Maglinant peripheral nerve sheath tumor	1	1.5	28	28	1: 0
	Sarcoma with myofibroblastic differentiation	1	1.5	40	40	0: 1
	Spindle cell neoplasm (low grade)	1	1.5	44	44	0: 1
	Spindle cell sarcoma	1	1.5	38	38	1: 0

### Mutation spectrum in Asian sporadic sarcoma cohort

**Figure 1:**
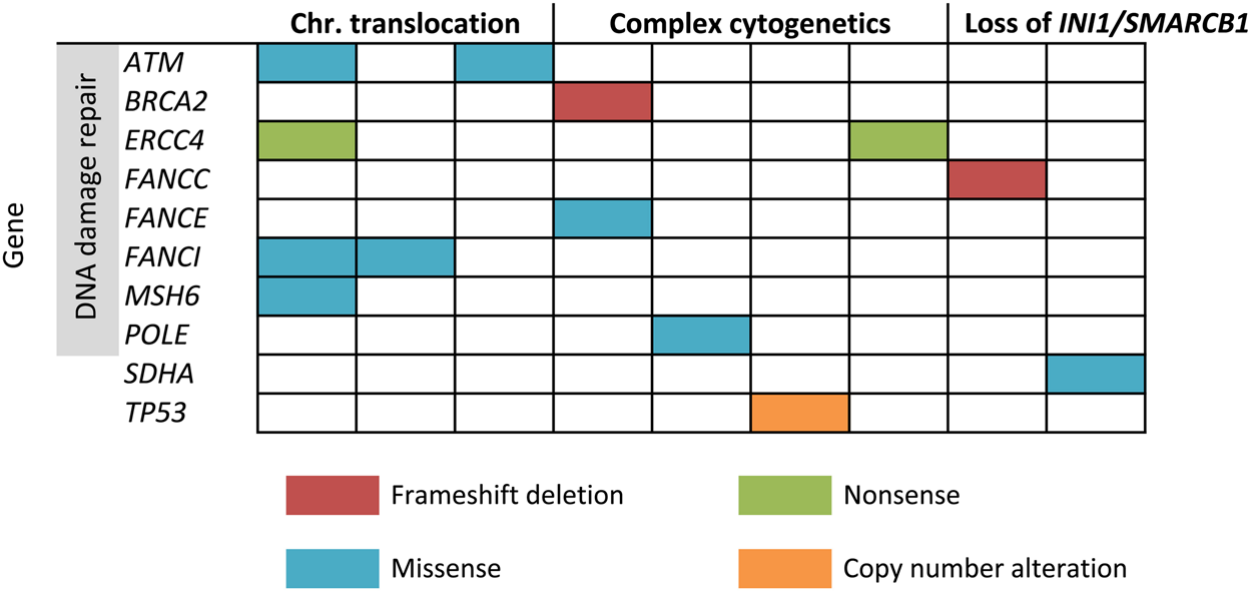
Predicted pathogenic germline variants found in the sporadic sarcoma cohort of this study. Diagram depicting distribution of the predicted pathogenic germline mutations across 10 cancer-associated genes according to three histological categories based on an arbitrary genetics-driven classification. Each column represents one patient. The mutation type is colour-coded as shown below the diagram.

Using our variant prioritization pipeline, we found 65 non-silent mutations, of which 32 (49.2%) were VUS, 20 (30.8%) benign and 13 (20.0%) were predicted to be pathogenic. Of the 13 predicted pathogenic mutations, 12 were identified by targeted sequencing, which comprised eight missense mutations, two nonsense and two frameshift mutations (Table 2). These mutations affected 9 genes, two each in *ATM, ERCC4* and *FANCI*, and one each in *BRCA2, FANCC, FANCE, MSH6*, *POLE*, and *SDHA*. One copy number alteration affecting *TP53* was detected through digitalMLPA (Table 2). Seven of these variants have been observed in very low frequencies in the East Asian population of 1000 G and ExAC databases, whereas the remaining variants are novel (Table 2). Of these mutations, 11 (84.6%) occurred in genes associated with DNA damage repair (DDR) and the remaining two (15.4%) in known cancer predisposition genes (Fig. 1).

**Table 2:** Predicted deleterious germline mutations across 10 genes identified from targeted genomic sequencing and digitalMLPA with clinical characteristics in the 9 patients.

Patient ID	Histology	Age at Dx (year)	Eth.	Sex	Gene	Mutation type	Nucleotide change	Protein change	Population frequency		ACMG class.	M-CAP score	M-CAP 95% sensitivity	Patient FHx
									1000G_EAS	ExAC_EAS				
S-032-NMM	Epitheloid sarcoma	24	OTH	F	*SDHA*	MS	c.1657G>A	p.Asp553Asn	0	0	VUS	0.262	PP	Aunt: Womb ca
S-039-THYA	GCTB	16	CH	F	*ERCC4*	NS	c.2169 C>A	p.Cys723*	0	0.06%	P	N.A.	N.A.	No record
S-073-SBB	LMS	49	MY	F	*TP53*	CNA	c.(?_1–230)_(118_177)del	-	novel	novel	P	N.A.	N.A.	No FHx of ca
S-104-SWK	Alveolar RMS	24	CH	F	*ATM*	MS	c.2770 C>T	p.Arg924Trp	0	0	VUS	0.253	PP	Uncle: NPC
					*ERCC4*	NS	c.2169 C>A	p.Cys723*	0	0.06%	P	N.A.	N.A.	
					*FANCI*	MS	c.1739A>G	p.Asn580Ser	novel	novel	VUS	0.044	PP	
					*MSH6*	MS	c.3851 C>T	p.Thr1284Met	0	0.03%	VUS	0.275	PP	
S-108-KYL	Epitheloid sarcoma	48	CH	F	*FANCC*	FS Del	c.1377_1378del	p.Ser459fs	novel	novel	LP	N.A.	N.A.	Father, Uncle, Grandfather: colon ca at 50’s/60’s; Sister: leukemia
S-110-SSK	LMS	40	CH	F	*POLE*	MS	c.2540 G>A	p.Arg847Gln	0.2%	0.13%	VUS	0.019	LB	No FHx of ca
S-112-SLK	Synovial sarcoma	45	CH	M	*FANCI*	MS	c.2183 A>G	p.Asp728Gly	0	0.06%	VUS	0.029	PP	No FHx of ca
S-114-YKC	Synovial sarcoma	49	CH	F	*ATM*	MS	c.512 A>G	p.Tyr171Cys	novel	novel	VUS	0.106	PP	No record
S-140-LTV	UPS	48	OTH	F	*BRCA2*	FS Del	c.1341_1342del	p.Pro447fs	novel	novel	LP	N.A.	N.A.	No FHx of ca
					*FANCE*	MS	c.1342 G>A	p.Glu448Lys	novel	novel	VUS	0.037	PP	

Abbrevation: ca, cancer; CH, Chinese; CNA, copy number alteration; Dx, diagnosis; Eth., ethnicity; F, female; FHx, family history; freq., frequency; FS Del, frameshift deletion; GCTB, giant cell tumor of bone; LMS, leiomyosarcoma; LB, likely benign; LP, likely pathogenic; M, male; MS, missense; MY, Malay; N.A., not available; NPC, nasopharyngeal cancer; NS, nonsense; OTH, other; P, pathogenic; Pop’n, population; PP, possibly pathogenic; RMS, rhabdomyosarcoma; UPS, undifferentiated pleomorphic sarcoma; VUS, variant of uncertain significance.

The predicted pathogenic mutations were found in 9 patients (13.6%, 95% CI: 6.8–24.8%) across 10 genes (Table 2). A recent study on germline mutations in pediatric cancers compared the prevalence of 60 autosomal-dominant (AD) genes in their cancer cohort with the 1000 G population^9^. Using their 1000 G data, we repeated the comparison against our cohort on the subset of our genes that overlapped with their 60 AD genes and observed a prevalence of predicted pathogenic mutation carriers at 6.1%, which is significantly higher than 1.1% in the 1000 G population (Fisher’s exact test, *P* = 0.01) (Supplementary Table S1).

Of the 13 mutations predicted pathogenic from our pipeline, five met the criteria for pathogenic/likely pathogenic classification recommended by the American College of Medical Genetics (ACMG) guidelines^12^ (Table 2, Supplementary Table S2). These include the two frameshift mutations in *BRCA2* and *FANCC*, two *ERCC4* nonsense mutations and one *TP53* copy number alteration. Additionally, three predicted pathogenic mutations are recommended by ACMG for return as incidental findings to patients^13^: one missense mutation in *MSH6*, one *BRCA2* frameshift deletion and one *TP53* copy number alteration. These alterations occurred in three patients of differing sarcoma histologies (Table 2). Age at sarcoma diagnosis was ≥45 years in all patients except the *MSH6* mutation carrier, whose age-at-diagnosis was 24 years old.

### Mutations in DNA damage repair (DDR) genes

**Figure 2:**
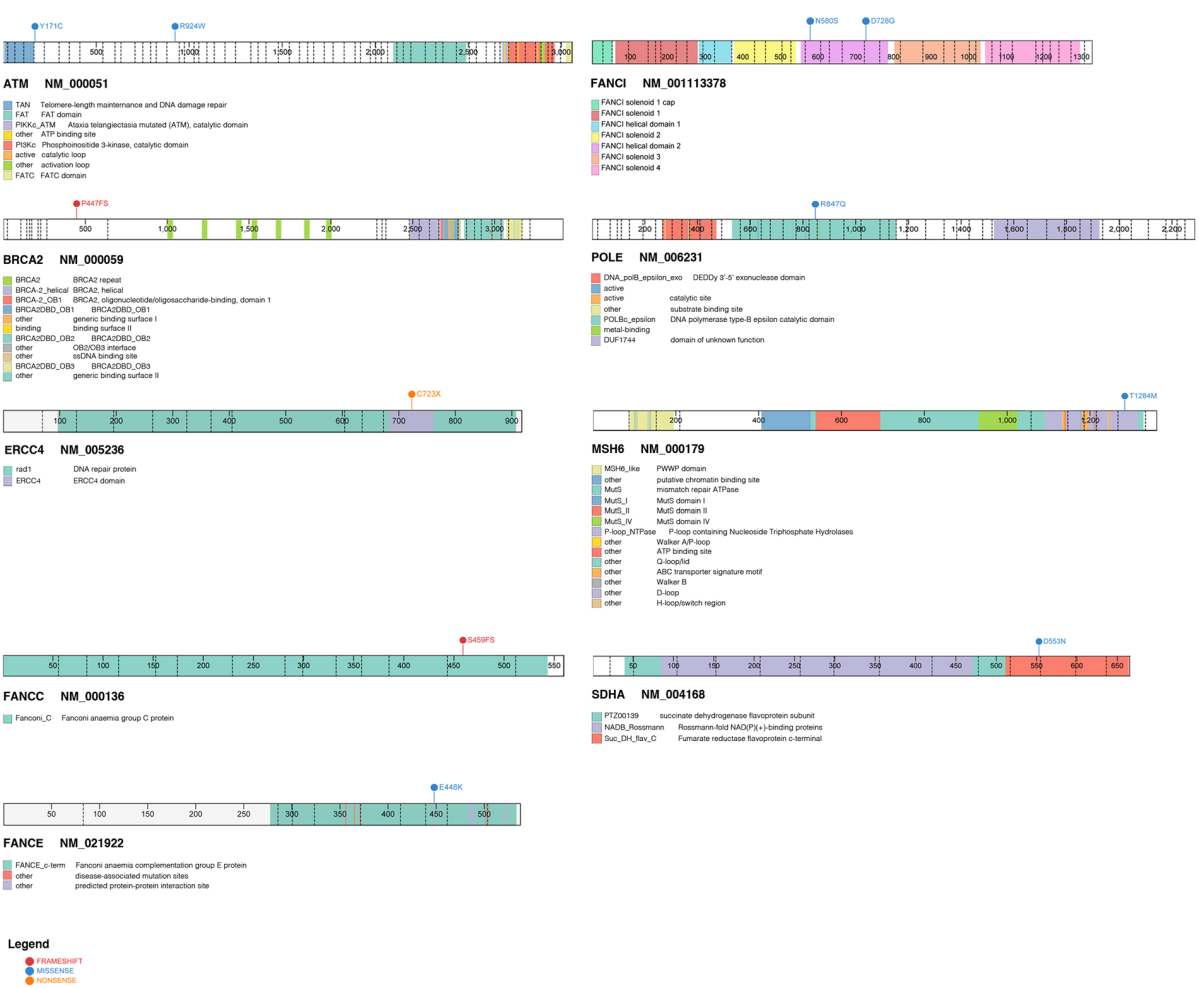
Visualization of protein domains for the genes with predicted pathogenic mutations identified in this study. *TP53* with copy number alteration is not visualized.

Among the 11predicted pathogenic mutations detected in DDR genes, two were frameshift deletions, two nonsense and seven missense mutations (Table 2). Eight DDR genes were affected; *ATM, BRCA2*, *ERCC4*, *FANCC, FANCE, FANCI, MSH6*, and *POLE* (Fig. 1). Truncating mutations, including frameshift deletions and nonsense mutations, occurred in *BRCA2, FANCC* and *ERCC4*. The *ERCC4* nonsense mutation (p.Cys723*) was found in two patients, who were diagnosed with giant cell tumor of bone and alveolar rhabdomyosarcoma at 16- and 24-years-old respectively (Table 2). This mutation occurred within ERCC4 domain (Fig. 2), which is the nuclease catalytic site of ERCC4^14^. Some of the remaining DDR mutations were also mapped to functionally important protein domains (Fig. 2); for instance the TAN (Tel1/ATM N-terminal) domain of ATM and the catalytic domain of POLE.

Seven patients harbored the 11predicted pathogenic mutations, marking a germline DDR mutation carrier frequency of 10.6% (95% CI: 4.7–21.2%) within our cohort. The mutations were not observed to be associated with any particular sarcoma histology. However, two patients were found to harbour multiple germline mutations in DDR genes; both were female, one with alveolar rhabdomyosarcoma at 24-years-old and the other had undifferentiated pleomorphic sarcoma at age 48 years (Supplementary Table S3). Interestingly, the former carried four germline mutations, all affecting DDR genes: *ATM, ERCC4, FANCI* and *MSH6*. A review of her family history revealed an uncle with nasopharyngeal cancer. Unfortunately, we were not able to reach the patient for more detailed familial information nor were we able to establish the somatic status of these variants as her tumor specimen was unavailable. In the latter patient with UPS, sequencing of her tumor showed loss of heterozygosity of the *BRCA2* variant, supporting the pathogenic prediction of this variant (Supplementary Figure S4).

### Mutations in other known cancer predisposition genes

Through targeted sequencing, one predicted pathogenic missense mutation was identified in *SDHA*, a known cancer predisposition gene, in a patient diagnosed with epitheloid sarcoma at 24-years-old (Table 2, Fig. 1). The mutation mapped to the fumarate reductase C-terminal of SDHA (Fig. 2), a catalytic domain in which germline mutations have been reported to be deleterious in patients presenting paragangliomas and pheochromocytomas as well as Leigh syndrome^15–17^. Additionally, digitalMLPA revealed a gross deletion of *TP53* exon 1 in a female patient with leiomyosarcoma diagnosed at 49-years-old and no records of familial cancer (Table 2). Validation by quantitative PCR (qPCR) confirmed the heterogeneous germline deletion and the loss of heterozygosity at this site in the patient tumor (Supplementary Figure S4).

### Association of mutations with sarcoma histology and family history

The nine patients carrying predicted pathogenic mutations had varying sarcoma histological diagnoses (Table 2). To explore potential associations between affected genes and tumor spectrum, the varying histological subtypes were categorized into three arbitrarily-assigned genetics-driven classifications based on literature: chromosomal translocation, complex genetics and loss of *INI1/SMARCB1* (Supplementary Table S5); however, we did not find any clear associations between mutated genes and histological subtypes (Fig. 1). We also assessed for potential correlation of the predicted pathogenic germline mutations with family histories. Clinical records of these patients were revisited to determine any family history that may have been missed by the treating clinician, however no evident correlation observed (Table 2), suggesting that family history should not be the sole inclusion factor when considering genetic predisposition in sarcoma patients.

### Variants of uncertain significance

In our analysis, a total of 32 VUS (Fig. 1) occurred across 20 genes in 25 patients (Supplementary Table S6). Amongst the 66 patients, 19 (28.8%) carry at least one VUS, and 5 (7.6%) had two VUS and 1 (1.5%) had three VUS (Supplementary Fig. S7). *BRIP1* had the most VUS per gene with four VUS, followed by *BRCA1* and *RAD50* with three VUS each, and two VUS each for the genes *FANCC, FANCI*, *MRE11A, MSH2* and *RET*. Of the VUS, 24 (75.0%) occur in DDR genes, closely reflecting the enrichment observed in the predicted pathogenic variants.

## Discussion

To our knowledge, our study is the first to screen for germline cancer gene mutations in Southeast Asian sporadic sarcoma patients. Whereas the ISKS^7^ recently indicated that a large proportion of sarcomas may harbor a germline component, our study differs in that our cohort was prospectively recruited solely on the basis of young age at diagnosis (<50 years). Taken together with the ISKS findings, our study independently confirms in an entirely Asian cohort that a substantial fraction of apparently sporadic sarcomas may harbour a germline component.

In our cohort of 66 sarcoma patients, 13.6% (95% CI: 6.8–24.8%) had at least one predicted pathogenic germline mutation in the 52 cancer-associated gene panel. Although lower than the 55% reported in the ISKS^7^, this can be potentially explained by a combination of factors. First, the ISKS gene panel is larger than ours (72 vs 52 genes). Second, while family history is not an inclusion criteria for the ISKS, patients with suspicious family histories may be more likely to be referred to the study. This is consistent with 17% of informative families in ISKS meeting the criteria for recognized cancer syndromes.

For this study, we used a local database of germline variants detected in a healthy matched cohort, which allowed us to remove 16 candidate variants that are probably rare, population-specific polymorphisms not found in databases such as ExAC and 1000 G. This illustrates the importance of having an ancestry-matched cohort of decent size to filter rare polymorphisms, echoing the recent findings where by African-American patients had variants misclassified as pathogenic but were subsequently reclassified as benign in light of additional population data^18^.

Half of the predicted pathogenic variants identified in this study are novel, and these mutations mostly affect DDR genes. Interestingly, a truncating mutation in *ERCC4* (p.Cys723*) was found in two patients with sarcoma diagnosed under age 25 years (Table 2). Apart from playing a key role in DDR, *ERCC4* is also involved in maintaining genomic stability^19^. This truncating mutation has been observed in gastric cancer tumors, and was shown to impair DNA repair capacity in CHO-K1 cells^20^. Incidentally, the ISKS reported an excess of pathogenic variants in *ERCC2*. The observation of *ERCC2* and *ERCC4* predicted pathogenic mutations in our study and the ISKS, coupled with the early age-of-onset in our two patients, suggests a potential role for the nucleotide excision repair (NER) pathway in sarcoma predisposition.

The prevalence of predicted pathogenic DDR gene mutation carriers in our cohort (10.6%) suggests that constitutional defects in this pathway may be associated with sarcoma. This is consistent with the enrichment of pathogenic mutations in DDR-related genes such as *ATM* and *BRCA2* seen in the ISKS^7^. Double-stranded DDR is highly conserved and crucial for chromosome structure maintenance and genomic stability. From our analysis of TCGA sarcoma data^21^ for pathogenic somatic mutations in these eight DDR genes, we observed a 3.4% prevalence, suggesting that these genes may indeed have a role in sarcomagenesis. It is also noteworthy that only one patient in our cohort harboured a germline *TP53* deletion, consistent with the relatively lower prevalence of germline^6^ versus somatic^21–23^ *TP53* mutations in sarcomas.

The presence of multiple predicted pathogenic DDR gene germline mutations (*ERCC4, ATM, FANCI, MSH6*) in an early-onset sarcoma in our study suggests that multiple pathogenic mutations may have an additive effect towards sarcoma predisposition, a hypothesis supported by the ISKS in which an earlier age-at-diagnosis was correlated with the cumulative burden of multiple pathogenic mutations^7^. Notably, all the variants found in our two patients with multiple predicted pathogenic germline mutations occurred in DDR genes (Table 2). Both patients have one protein-truncating variant co-occuring with predicted pathogenic single nucleotide variants. It is conceivable that even if the deleterious effect of each mutation is non-significant independently, the collective impact of these co-occurring predicted pathogenic mutations may potentially lead to impaired DNA repair and genomic instability, therefore conferring susceptibility to tumorigenesis. Recent findings showing frequent germline mutations in DNA homologous recombination genes within a metastatic prostate cancer cohort suggests the potential application of targeted therapies, such as PARP1-inhibition and platinum-based chemotherapy^24^. The excess of predicted pathogenic DDR gene germline mutations in our sporadic sarcoma cohort suggests that a subset of sarcomas may be candidates for such targeted therapies.

Several limitations were encountered in this study. First, the cohort size is constrained by the rarity of sarcomas. Second, heterogeneous histology and sparse patient family history precluded any associations with their genotype. Third, the performance of various *in silico* variant pathogenicity prediction algorithms can be variable, and there remains no consensus on the choice of algorithms for predicting variant pathogenicity^12^. Thus, interpretation of disease causality for variants, especially missense variants, remains a challenge despite proposed guidelines^12, 25^. We sequenced tumors of the patients harbouring the missense variants as a means of assessing pathogenicity but tumor DNA was not available for most of the patients, hence the missense variants of these patients were interpreted with caution. The only two variants we successfully validated – *FANCE* (p.Glu448Lys) and *FANCI* (p.Asp728Gly) – did not show loss of heterozygosity, however structural data has shown that these positions of the two *FANC* genes are involved in the important protein-protein interaction with FANCD2^26, 27^. These genes are members of the Fanconi anemia (FA) pathway, which is known to predispose to FA and other malignancies when impaired^28, 29^. FANCE has been demonstrated to play a key role in the architecture of the FA core complex by mediating interactions with FANCD2^30, 31^ whereas FANCI forms a heterodimer with FANCD2 known as the ID complex^26^, both of which are critical for the activity of the FA pathway^28^. As the Glu448 residue of FANCE is highly conserved across species and important for FANCD2 binding^27^, mutation of Glu448Lys is likely to impact on the interaction between FANCE and FANCD2 due to the change in residue size and charge. The two *FANCI* variants seen in our cohort – Asp728Gly and Asn580Ser – corresponded to residues in the FANCI helical domain 2 that are highly conserved across species^26^. In particular, Asn580 is located in a region concentrated with polar residues shown to interface with FANCD2. While the specific effect of these mutations remains to be functionally confirmed, the potential deleterious consequence on the activity of FANCD2 in addition to the functional studies reported in literature demonstrating the loss of protein function in the *FANC*-family genes^32, 33^ collectively provide some evidence favouring the assignment of pathogenicity to the missense mutations we observed in this study. Importantly, the consistency of our findings with a larger, more powered study such as the ISKS indicates that our bioinformatics approach can reasonably discover potentially pathogenic germline mutations in our cohort. Despite these limitations, our findings show that a considerable proportion of sporadic sarcomas may have underlying genetic predisposition.

In summary, our study is the first to investigate and identify an excess of potentially pathogenic germline mutations in a Southeast Asian cohort of young sarcoma. Our findings, together with that of the ISKS, show that prevalence of pathogenic germline mutation carriers in an apparently sporadic sarcoma cohort may be higher than anticipated and that sarcoma has a significant hereditary component. Additionally, frequent observation of potentially pathogenic germline mutations in the DDR pathway suggest that inherited defects in this pathway may contribute to sarcoma predisposition. Sarcoma patients encountered in the clinic, especially young ones, should therefore be treated as potential carriers of germline pathogenic mutations in cancer predisposition genes regardless of family history and considered for genetic testing. Insights from this study will help direct further efforts to enhance our understanding of genetic predisposition in sarcoma with potentially significant impact on patient-care.

## Materials and Methods

### Patients

Patients consulted at our sarcoma subspecialty clinic at the National Cancer Centre Singapore were prospectively recruited for this study. Sixty-six patients under age 50 years of varying sarcoma subtypes (excluding GIST) were selected for sequencing. Patient clinical data including sarcoma histology, personal and family history of cancer were collated (Table 1). Patient-derived peripheral blood was used to obtain genomic DNA for sequencing. This study was approved by the SingHealth Centralised Institutional Review Board (IRB 2010/426/B) with signed informed consent from all patients. All study procedures were carried out in accordance with the approved guidelines.

### Targeted genomic sequencing

A panel of 52 genes associated with cancer-predisposition and DNA damage repair was customized using Agilent SureDesign (Agilent, Santa Clara, CA, USA). Purified patient genomic DNA were sheared to 150–200 base pairs (bp) fragments for targeted capture of the customized gene panel. Captured libraries were pooled and sequenced on Illumina Hiseq. 4000 (Illumina Inc., San Diego, CA, USA) to an average depth of 988X, with 87% of target bases covered > 20X. Methods are detailed under Supplementary Methods.

### Variant prioritization pipeline

Sequenced reads were aligned to the human reference genome (hs37d5) as detailed in Supplementary Methods. Missense variants and micro-indels were identified, then filtered by read-depth and quality score. Variants overlapping target regions were retained for analysis. To prioritize candidate germline variants, filtered variants were annotated and common polymorphisms removed by excluding variants present in >1% of East-Asian or South-Asian population as defined by Exome Aggregation Consortium (ExAC) and 1000 Genomes (1000 G) databases^34, 35^. Variants found using an in-house database of common polymorphisms in our local population (n = 454) were excluded, then filtered to retain only splice-site and non-synonymous exonic variants. Frameshift, nonsense and splice-site variants were deemed pathogenic. Missense variants were classified as potentially pathogenic, variant of uncertain significance (VUS) or benign using *in silico* prediction algorithms SIFT, PolyPhen2 HDIV, Mutation Assessor, FATHMM and CADD. Variants were considered potentially pathogenic if ≥3 algorithms predicted the variant to be damaging, and benign if none considered the variant damaging. Remaining variants were categorized as VUS. Analysed sequencing data were deposited in the European Nucleotide Archive (accession no. PRJEB20843). Variant predictions were checked on InterVar^36^ for interpretation based upon the American College of Medical Genomics and Genetics (ACMG) guidelines^12^ and validated against M-CAP (Mendelian Clinically Applicable Pathogenicity), a recently-published clinical pathogenicity classifier with improved sensitivity^37^. Pathway analysis was performed using Molecular Signatures Database^38^ (MSigDB; Broad Institute). Protein domains were visualized using ProteinPaint^39^ (PeCan Data Portal, https://pecan.stjude.org/proteinpaint/).

### DigitalMLPA analysis

Patient genomic DNA were hybridized to a mixture of probes targeting 29 hereditary cancer genes, ligated, barcoded and subsequently sequenced on Illumina MiSeq (Illumina Inc., San Diego, CA, USA). Data were analyzed in collaboration with the manufacturer using a pre-release version of Coffalyser.Net (MRC-Holland, Amsterdam, The Netherlands). For detailed methods, refer to Supplementary Methods.

### Validation of variants

Candidate variants were validated by Sanger sequencing using BigDye Terminator v3.1 (ABI, ThermoFisher Scientific Corporation). Resulting chromatograms were analyzed using Mutation Surveyor (Softgenetics, PA, USA). Copy number variants detected through digitalMLPA were validated by quantitative PCR (qPCR). Cycle threshold (C_t_) values were normalized to GAPDH endogenous control and fold-change in gene dosage was calculated using the ΔΔC_t_ method by normalizing against a pool of three healthy controls. For validation of the somatic status of candidate variants, Sanger sequencing was performed on tumor DNA extracted from fresh frozen or formalin-fixed paraffin embedded tumors using QIAamp DNA mini (Qiagen, 51304) or QIAamp FFPE tissue (Qiagen, 56404) kits.

### Statistical analyses

Patient characteristics and sequencing results were tabulated with descriptive statistics including medians, means and standard deviations for proportions with 95% confidence interval (CI). Proportions were analyzed using Fisher’s exact test. All *P*-values are two-tailed.

### Analysis of The Cancer Genomic Atlas (TCGA) mutations

Somatic mutations from TCGA sarcoma study^21^ were downloaded from the Broad Genome Data Analysis Centre (GDAC) portal (Broad Institute, http://firebrowse.org), annotated and filtered as described above.

## Electronic supplementary material

Supplementary Material
