# Supplementary material for: Germline Mutations in Cancer Predisposition Genes are Frequent in Sporadic Sarcomas
Source: Sci Rep. 2017 Sep 6;7:10660. doi: 10.1038/s41598-017-10333-x (PMC5587568; doi:10.1038/s41598-017-10333-x)
Supplement: Supplementary file 1 — Supplementary Material [file 41598_2017_10333_MOESM1_ESM.pdf]

SUPPLEMENTARY MATERIAL

Germline Mutations in Cancer Predisposition Genes are Frequent in Sporadic Sarcomas

Sock Hoai Chan, PhD[1#], Weng Khong Lim, PhD[2,3#], Nur Diana Binte Ishak, BS[1], Shao-Tzu Li, MS LCGC[1], Wei Lin Goh, BS[4], Gek San Tan, BS[5], Kiat Hon Lim, MD[5,6], Melissa Teo, MD[7], Cedric Ng Chuan Young, MS[8], Simeen Malik, PhD[3,9], Mann Hong Tan, MD[10], Jonathan Yi Hui The, MD[11], Francis Kuok Choon Chin, MD[11], Sittampalam Kesavan, MD[6], Sathiyamoorthy Selvarajan, MD[6], Patrick Tan, MD PhD[3,9,12,13], Bin Tean Teh, MD PhD[8,9,12,14], Khee Chee Soo, MD[7], Mohamad Farid, MD[4], Richard Quek, MD[4,15*], Joanne Ngeow, MD MPH[1,15*]

[1] Cancer Genetics Service, Division of Medical Oncology, National Cancer Centre Singapore, Singapore 169610

[2] Centre for Computational Biology, Duke-NUS Medical School Singapore, Singapore 169857

[3] Singhealth Duke-NUS Institute of Precision Medicine (PRISM), Singapore 169856

[4] Division of Medical Oncology, National Cancer Centre Singapore, Singapore 169610

[5] Department of Molecular Pathology, Singapore General Hospital, Singapore 169610

[6] Department of Anatomical Pathology, Singapore General Hospital, Singapore 169610

[7] Division of Surgical Oncology, National Cancer Centre Singapore, Singapore 169610

[8] Laboratory of Cancer Epigenome, Division of Medical Sciences, National Cancer Centre Singapore, Singapore 169610

[9] Cancer & Stem Cell Biology Program, Duke-NUS Medical School Singapore, Singapore 169857

[10] Department of Orthopaedic Surgery, Singapore General Hospital, Singapore 169610

[11] Division of Radiation Oncology, National Cancer Centre Singapore, Singapore 169610

[12] Cancer Science Institute of Singapore, National University Singapore, Singapore 117599

[13] Division of Molecular and Cellular Research, National Cancer Centre Singapore, Singapore 169610

[14] Institute of Molecular and Cellular Biology, A*STAR, Singapore 138673

[15] Oncology Academic Clinical Program, Duke-NUS Medical School Singapore, Singapore 169857

[#]Joint First Authors

[*]Joint Senior Authors

Correspondence and reprint requests to:

Dr Joanne Ngeow

Cancer Genetics Service, Division of Medical Oncology, National Cancer Centre Singapore, 11 Hospital Drive, Singapore 169610. Tel: +65 6436 8172. Email: Joanne.Ngeow.Y.Y@singhealth.com.sg.

OR

Dr Richard Quek

Division of Medical Oncology, National Cancer Centre Singapore, 11 Hospital Drive, Singapore 169610. Tel: +65 6436 8000. Email: Richard.quek.h.h@singhealth.com.sg.

SUPPLEMENTARY METHODS

Targeted genomic sequencing

Agilent SureDesign (Agilent, Santa Clara, USA) was used to customized a panel of 52 cancer-predisposition and DNA damage repair genes, including *APC, ATM, ATR, BLM, BMPR1A, BRCA1, BRCA2, BRIP1, CDH1, CDK4, CDKN2A, CHEK2, EPCAM, ERCC4, FANCA, FANCB, FANCC, FANCD2, FANCE, FANCF, FANCG, FANCI, FANCL, FANCM, MEN1, MET, MLH1, MRE11A, MSH2, MSH6, MUTYH, PALB2, PDGFRA, PMS1, PMS2, POLD1, POLE, RAD50, RAD51C, RAD51D, RET, SDHA, SDHAF2, SDHB, SDHC, SDHD, SMAD4, STK11, TP53, VHL, WRN* and *XRCC2.* Patient genomic DNA from peripheral blood was purified using Blood and Cell Culture kit (Qiagen, 13343) according to manufacturer's protocol and quantified using Qubit dsDNA HS-Assay Kit (Life Technologies, Q32851). 100 nanograms (ng) of DNA per patient were sheared to fragments of 150-200 base pairs (bp) on Covaris-E220 sonicator (Woburn, MA, USA). Targeted capture of the customized panel was performed using SureSelect XT2 Target Enrichment kit (Agilent, G9621A) and the indexed captured DNA were quantified on Agilent Bioanalyzer with Agilent High-Sensitivity kit (Agilent, 5067). Libraries were pooled for sequencing on Illumina Hiseq4000 (Illumina Inc., San Diego, CA, USA) using paired-end 100 bp reads.

Bioinformatics analysis

Sequenced reads were aligned to the human reference genome (hs37d5) using Burrows-Wheeler Aligner (BWA) version 0.7.10 [1]. Duplicate read marking and read sorting were performed using SAMBLASTER version 0.1.22 [2] and Sambamba version 0.5.4 [3] respectively. Missense variants and microindels were identified using Freebayes version 0.9.21. Variants were filtered by read depth (>100X) and variant quality score (Phred score > 30). Variants were annotated using

wANNOVAR [4] web application (http://wannovar.usc.edu) and then filtered to remove common polymorphisms present in 1% or more of East Asian or South Asian population defined by Exome Aggregation Consortium (ExAC) and 1000 Genomes (1000G) databases [5, 6]. Additionally, variants were checked against our in-house database to remove common polymorphisms present in our local population. Only splice-site and nonsynonymous exonic variants were retained for further analysis. Candidate germline variants were prioritized based on the following criteria. Frameshift, nonsense and splice-site variants are deemed pathogenic. Missense variants were categorized as pathogenic, variant of uncertain significance (VUS) or benign based on the collective predictions of five *in silico* algorithms: SIFT, PolyPhen2 HDIV, Mutation Assessor, FATHMM and CADD. Variants were prioritized as probable pathogenic if scored as damaging or probably damaging by three or more algorithms, and benign if none of the algorithms scored the variant as damaging. All remaining variants were classified as VUS. Candidate variants were visually inspected using Integrative Genomics Viewer (IGV; Broad Institute). Pathway analysis of the genes was performed using Molecular Signatures Database (MSigDB; Broad Institute) against gene sets from Reactome, Pathway Interaction Database, and KEGG [7–12].

Digital Multiplex Ligation-dependent Probe Amplification (digitalMLPA) analysis

Multiplex ligation-dependent probe amplification (MLPA) was performed on purified patient genomic DNA using digitalMLPA probe mix D001-X1 Hereditary Cancer Panel-1 and SALSA digitalMLPA reagent DRK01-IL kits (MRC Holland, Amsterdam, The Netherlands) according to manufacturer’s instructions. Genomic DNA were hybridized to a mixture of probes targeted to 29 hereditary cancer genes including *APC, ATM, BARD1, BAP1, BMPR1, BRCA1, BRCA2,*

BRIP1, CDKN2A, CDK4, CDH1, CHEK2, EPCAM, MUTYH, MSH2, MSH6, MLH1, MITF, NBN, *PMS2*, *PTEN*, *POLE*, *PALB2*, *RAD51C, RAD51D, SCG5/GREM1, SMAD4, STK11*, *TP53*. Hybridized probes were ligated and a specific barcode was incorporated into each patient sample. Ligated probes were subsequently amplified by polymerase chain reaction (PCR) and sequenced on Illumina MiSeq (Illumina Inc., San Diego, CA, USA).

Validation of candidate variants

Candidate variants from targeted genomic sequencing were validated by Sanger sequencing. Primers flanking each variant were designed on Primer3 [13, 14], PCR amplified busing PlatinumTaq DNA polymerase (Invitrogen, 10966) and then sequenced using BigDye Terminator v3.1 (ABI, ThermoFisher Scientific Corporation) on 3130xl Genetic Analyzer (ABI). Resulting chromatograms were analyzed using Mutation Surveyor software (Softgenetics, PA, USA). Copy number variants detected through digitalMLPA were validated by quantitative PCR (qPCR). Primers within the deleted regions were designed and qPCR performed using Ssofast Evagreen Supermix (Bio-rad, 172-5200) on CFX96 Real-Time PCR Detection System (Bio-rad) with the following conditions: 30 seconds (s) at 98°C for enzyme activation, followed by 40 cycles of 5 s each at 98°C and 58°C for denaturation and extension, completed with melt-curve analysis at 65°C-95°C. Cycle threshold (C_t) values were normalized to GAPDH endogenous control and fold change in gene dosage was calculated using the $\Delta\Delta C_t$ method by normalizing against a pool of three healthy controls. For validation of the somatic status of candidate variants, Sanger sequencing was performed on tumor DNA extracted from fresh frozen or formalin-fixed paraffin embedded tumors using QIAamp DNA mini (Qiagen, 51304) or QIAamp FFPE tissue (Qiagen, 56404) kits.

SUPPLEMENTARY TABLES AND FIGURES

Supplementary Table S1 : Comparison of mutation frequency of the known cancer predisposition genes in this study with the 1000G control dataset presented by Zhang *et. al* [15]. Abbrevation: No., number.

		**Current study (n=66)**	**1000G (n=966)**		
		No. mutation carriers	No. mutation carriers		
**Overlapping genes in panel**	*APC*	0	1		
	BMPR1A	0	0		
	BRCA1	0	1		
	BRCA2	1	4		
	CDH1	0	0		
	CDK4	0	0		
	CDKN2A	0	0		
	EPCAM	0	0		
	MEN1	0	0		
	MLH1	0	0		
	MSH2	0	0		
	MSH6	1	1		
	PALB2	0	0		
	PMS2	0	0		
	RET	0	0		
	SDHA	1	1		
	SDHAF2	0	0		
	SDHB	0	1		
	SDHC	0	0		
	SDHD	0	0		
	SMAD4	0	0		
	STK11	0	0		
	TP53	1	2		
	VHL	0	0		
				**Fisher's exact test**	
				p value	Odds ratio
**Total mutation carriers**		4 (6.1 %)	11 (1.1 %)	0.01	5.6

Supplementary Table S2 : Classification of the 13 germline variants based on the criteria outlined under the American College of Medical Genetics (ACMG) guidelines [16]. Abbrevation: CNA, copy number alteration; FS Del, frameshift deletion; LP, likely pathogenic; MS, missense; NS, nonsense; P, pathogenic; US, uncertain significance; VUS, variant of uncertain significance. Assignment for each criteria follows the guidelines published by Richards *et. al* [16].

**Gene**	**Mutation type**	**RefSeq transcript**	**Nucleotide change**	**Protein change**	**Population data**	**Computational and predictive data**	**Functional data**	**Classification**
ATM	MS	NM_000051	c.512A>G	p.Tyr171Cys	PM2			VUS
ATM	MS	NM_000051	c.2770C>T	p.Arg924Trp		US		VUS
BRCA2	FS Del	NM_000059	c.1341_1342del	p.Pro447fs	PM2	PVS1		LP
ERCC4	NS	NM_005236	c.2169C>A	p.Cys723*		PVS1, PM4	PS3[17]	P
ERCC4	NS	NM_005236	c.2169C>A	p.Cys723*		PVS1, PM4	PS3[17]	P
FANCC	FS Del	NM_000136	c.1377_1378del	p.Ser459fs	PM2	PVS1		LP
FANCE	MS	NM_021922	c.1342G>A	p.Glu448Lys	PM2	PP3		VUS
FANCI	MS	NM_001113378	c.1739A>G	p.Asn580Ser	PM2	US		VUS
FANCI	MS	NM_001113378	c.2183A>G	p.Asp728Gly		PP3		VUS
MSH6	MS	NM_000179	c.3851C>T	p.Thr1284Met	PM2	PP3		VUS
POLE	MS	NM_006231	c.2540G>A	p.Arg847Gln		PP3		VUS
SDHA	MS	NM_004168	c.1657G>A	p.Asp553Asn		PP3		VUS
TP53	CNA	NM_000546	c.(?_1-230)_(118_177)del	-	PM2	PVS1	PS3[18, 19]	P

Supplementary Table S3 : Germline mutations and the corresponding histology of the two patients with more than one predicted pathogenic germline mutation.

Patient ID	Histology	Age at diagnosis (year)	Sex	Affected gene	Nucleotide change	Protein change	Patient family history
S-104-SWK	Alveolar rhabdomyosarcoma	24	F	*ERCC4*<br>*ATM*<br>*FANCI*<br>*MSH6*	c.2169C>A<br>c.2770C>T<br>c.1739A>G<br>c.3851C>T	p.Cys723*<br>p.Arg924Trp<br>p.Asn580Ser<br>p.Thr1284Met	Uncle: nasopharyngeal cancer
S-140-LTV	Undifferentiated Pleomorphic Sarcoma	48	F	*BRCA2*<br>*FANCE*	c.1341_1342del<br>c.1342G>A	p.Pro447fs<br>p.Glu448Lys	No family history of cancer

Supplementary Figure S4 : Loss of heterozygosity (LOH) in tumors of two patients with deletion variants. (A) Validation of *TP53* exon 1 deletion in patient S-073-SBB by qPCR. Germline gDNA derived from patient peripheral blood (normal, patient) showed significant halving of the gene dosage ($p < 0.0001$) compared to healthy controls (normal, healthy). Patient tumor DNA (tumor, patient) confirmed LOH in the tumor. Values are represented as fold change of difference in genomic DNA copy number with reference to healthy control (n=3). All values are a mean of three replicate readings and error bars indicate standard error. P-values were computed using two-tailed Student's t-test. (B) Sanger trace of *BRCA2* frameshift deletion in patient S-140-LTV. Ref: reference sequence.

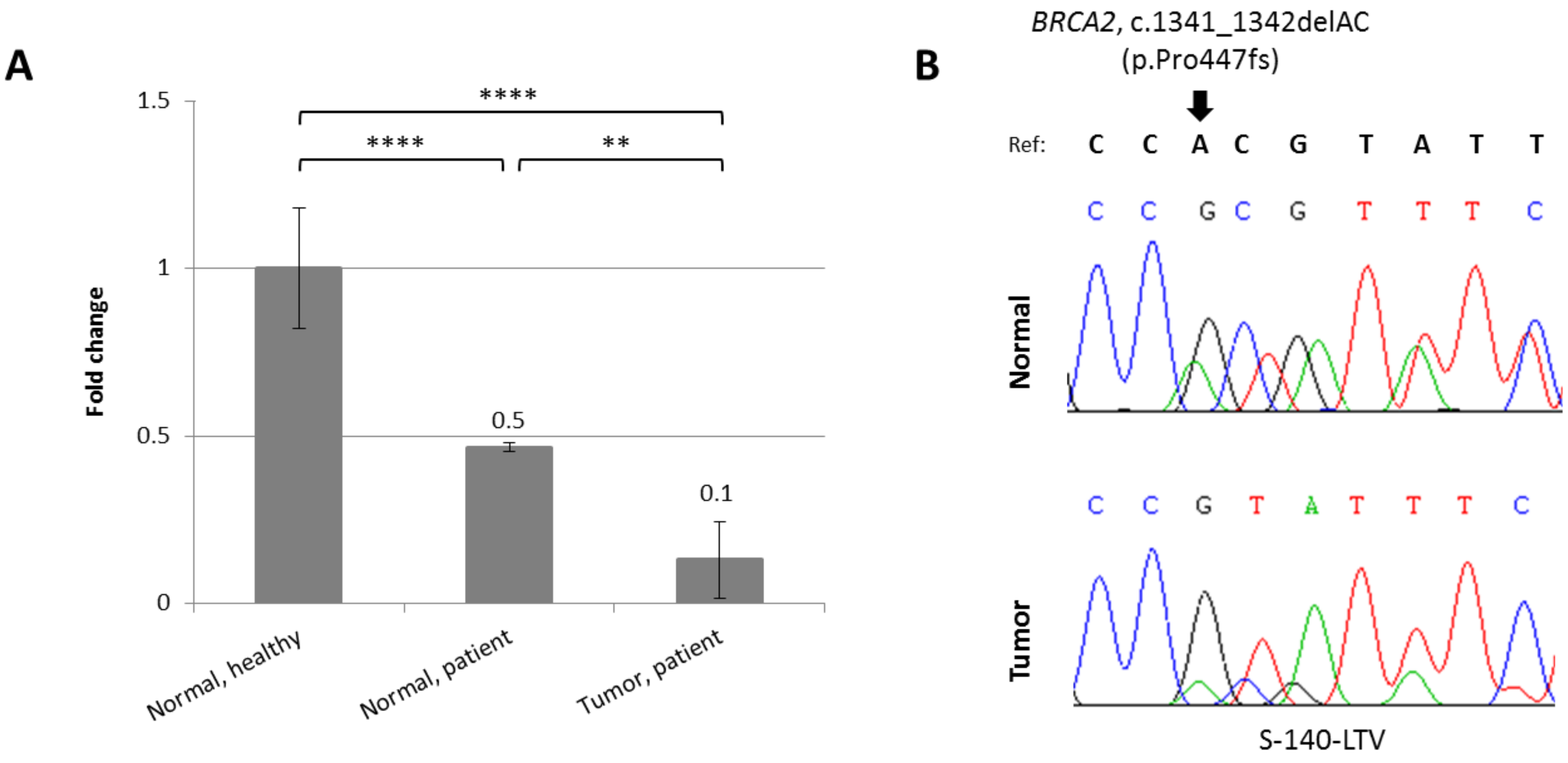

Supplementary Table S5 : The predicted pathogenic germline mutations listed by an arbitrary genetic-driven classification of patient histology.

Classification	Disease subtype	Sample ID	Affected gene	Nucleotide change	Protein change	Mutation type
Chromosomal translocation	Alveolar rhabdomyosarcoma	S-104-SWK	*ERCC4*	c.2169C>A	p.Cys723*	Nonsense
			ATM	c.2770C>T	p.Arg924Trp	Missense
			FANCI	c.1739A>G	p.Asn580Ser	Missense
			MSH6	c.3851C>T	p.Thr1284Met	Missense
	Synovial sarcoma	S-112-SLK	*FANCI*	c.2183A>G	p.Asp728Gly	Missense
		S-114-YKC	*ATM*	c.512A>G	p.Tyr171Cys	Missense
Complex cytogenetics	Undifferentiated Pleomorphic Sarcoma	S-140-LTV	*BRCA2*	c.1341_1342del	p.Pro447fs	Frameshift deletion
			FANCE	c.1342G>A	p.Glu448Lys	Missense
	Leiomyosarcoma	S-110-SSK	*POLE*	c.2540G>A	p.Arg847Gln	Missense
		S-073-SBB	*TP53*	c.(?_1-230)_(118_177)del	-	Copy number alteration
	Giant cell tumor of bone	S-039-THYA	*ERCC4*	c.2169C>A	p.Cys723*	Nonsense
Loss of *INI1/SMARCB1*	Epitheloid sarcoma	S-108-KYL	*FANCC*	c.1377_1378del	p.Ser459fs	Frameshift deletion
		S-032-NMM	*SDHA*	c.1657G>A	p.Asp553Asn	Missense

Supplementary Table S6 : Distribution of the 32 VUS identified across 20 genes in 25 patients. Twenty four of the VUS occurred in 13 DNA damage repair pathway genes.

Gene	No. VUS	DNA damage repair pathway
BRIP1	4	✓
BRCA2	3	✓
RAD50	3	✓
FANCC	2	✓
FANCI	2	✓
MRE11A	2	✓
MSH2	2	✓
RET	2	-
APC	1	-
BRCA1	1	✓
CHEK2	1	-
FANCA	1	✓
MET	1	-
MSH6	1	✓
MUTYH	1	✓
PALB2	1	✓
PMS1	1	✓
SDHA	1	-
SDHAF2	1	-
VHL	1	-

Supplementary Figure S7 : Frequency of VUS occurrence per patient within the sequenced cohort.

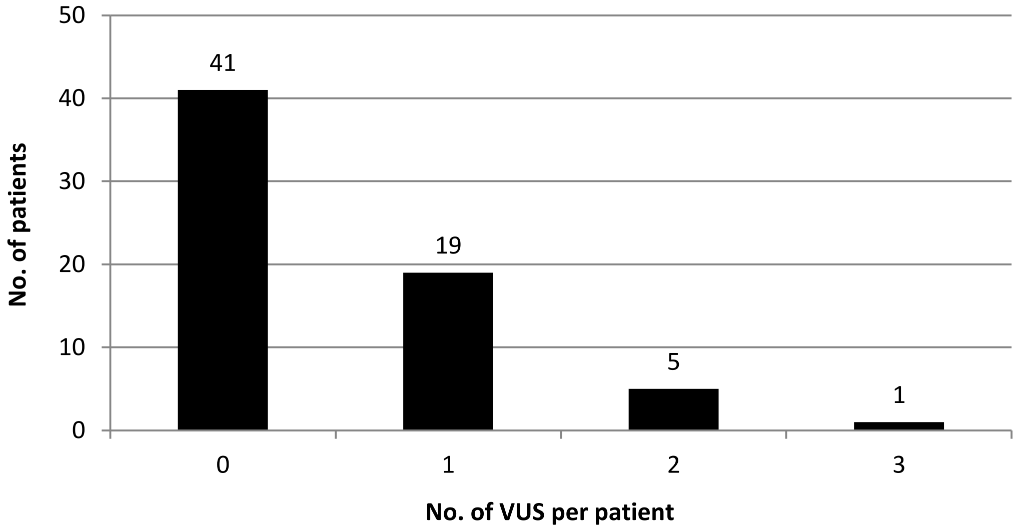

REFERENCES

1. Li H, Durbin R: Fast and accurate short read alignment with Burrows-Wheeler transform. Bioinforma Oxf Engl 25:1754–1760, 2009

2. Faust GG, Hall IM: SAMBLASTER: fast duplicate marking and structural variant read extraction. Bioinforma Oxf Engl 30:2503–2505, 2014

3. Tarasov A, Vilella AJ, Cuppen E, et al: Sambamba: fast processing of NGS alignment formats. Bioinformatics btv098, 2015

4. Wang K, Li M, Hakonarson H: ANNOVAR: functional annotation of genetic variants from high-throughput sequencing data. Nucleic Acids Res 38:e164–e164, 2010

5. Consortium T 1000 GP: An integrated map of genetic variation from 1,092 human genomes. Nature 491:56–65, 2012

6. Exome Aggregation Consortium (ExAC), Cambridge, MA [Internet]Available from: http://exac.broadinstitute.org

7. Subramanian A, Tamayo P, Mootha VK, et al: Gene set enrichment analysis: A knowledge-based approach for interpreting genome-wide expression profiles. Proc Natl Acad Sci 102:15545–15550, 2005

8. Croft D, Mundo AF, Haw R, et al: The Reactome pathway knowledgebase. Nucleic Acids Res 42:D472-477, 2014

9. Fabregat A, Sidiropoulos K, Garapati P, et al: The Reactome pathway Knowledgebase. Nucleic Acids Res 44:D481-487, 2016

10. Schaefer CF, Anthony K, Krupa S, et al: PID: the Pathway Interaction Database. Nucleic Acids Res 37:D674–D679, 2009

11. Kanehisa M, Sato Y, Kawashima M, et al: KEGG as a reference resource for gene and protein annotation. Nucleic Acids Res 44:D457-462, 2016

12. Kanehisa M, Goto S: KEGG: kyoto encyclopedia of genes and genomes. Nucleic Acids Res 28:27–30, 2000

13. Untergasser A, Cutcutache I, Koressaar T, et al: Primer3--new capabilities and interfaces. Nucleic Acids Res 40:e115, 2012

14. Koressaar T, Remm M: Enhancements and modifications of primer design program Primer3. Bioinforma Oxf Engl 23:1289–1291, 2007

15. Zhang J, Walsh MF, Wu G, et al: Germline Mutations in Predisposition Genes in Pediatric Cancer. N Engl J Med 373:2336–2346, 2015

16. Richards S, Aziz N, Bale S, et al: Standards and guidelines for the interpretation of sequence variants: a joint consensus recommendation of the American College of Medical Genetics and Genomics and the Association for Molecular Pathology. Genet Med 17:405–423, 2015

17. Wei Z-H, Guo W-H, Wu J, et al: A nonsense mutation in the Xeroderma pigmentosum complementation group F (XPF) gene is associated with gastric carcinogenesis. Gene 537:238–244, 2014

18. Khan D, Sharathchandra A, Ponnuswamy A, et al: Effect of a natural mutation in the 5′ untranslated region on the translational control of p53 mRNA. Oncogene 32:4148–4159, 2013

19. Chen J, Kastan MB: 5’-3’-UTR interactions regulate p53 mRNA translation and provide a target for modulating p53 induction after DNA damage. Genes Dev 24:2146–2156, 2010
